# Fast automated placement of polar hydrogen atoms in protein-ligand complexes

**DOI:** 10.1186/1758-2946-1-13

**Published:** 2009-08-12

**Authors:** Tobias Lippert, Matthias Rarey

**Affiliations:** 1 Center for Bioinformatics, 20146 Hamburg, Germany

## Abstract

**Background:**

Hydrogen bonds play a major role in the stabilization of protein-ligand complexes. The ability of a functional group to form them depends on the position of its hydrogen atoms. An accurate knowledge of the positions of hydrogen atoms in proteins is therefore important to correctly identify hydrogen bonds and their properties. The high mobility of hydrogen atoms introduces several degrees of freedom: Tautomeric states, where a hydrogen atom alters its binding partner, torsional changes where the position of the hydrogen atom is rotated around the last heavy-atom bond in a residue, and protonation states, where the number of hydrogen atoms at a functional group may change. Also, side-chain flips in glutamine and asparagine and histidine residues, which are common crystallographic ambiguities must be identified before structure-based calculations can be conducted.

**Results:**

We have implemented a method to determine the most probable hydrogen atom positions in a given protein-ligand complex. Optimality of hydrogen bond geometries is determined by an empirical scoring function which is used in molecular docking. This allows to evaluate protein-ligand interactions with an established model. Also, our method allows to resolve common crystallographic ambiguities such as as flipped amide groups and histidine residues. To ensure high speed, we make use of a dynamic programming approach.

**Conclusion:**

Our results were checked against selected high-resolution structures from an external dataset, for which the positions of the hydrogen atoms have been validated manually. The quality of our results is comparable to that of other programs, with the advantage of being fast enough to be applied on-the-fly for interactive usage or during score evaluation.

## Background

Pharmaceutical research focuses on finding novel ligands for proteins known to be disease-modifying. This research can be assisted by docking calculations which provide *in-silico *estimations of the binding mode and the binding affinity of putative ligand molecules and a protein [1]. Polar interactions, to which hydrogen bonds belong, play a major role in non-covalent protein-ligand interactions [2]. These hydrogen bonds have a direction which depends on the position of the involved hydrogen atoms. Hence their positions need to be known to correctly assess protein-ligand interactions with respect to their hydrogen bonds. However, they cannot be taken from the input structure but must be calculated for two reasons: First, as stated in the induced fit theory, the protein may respond to a bound ligand with changes in its conformation [3]. The energetically most simple change in conformation is a change in the position of the hydrogen atoms, resulting in a change of the active site's spatio-chemical properties. Second, protein structures may contain ambiguities that result from the experimental method, especially if it is X-ray crystallography, which has been used to determine most of the publicly available structures in the Protein Data Bank (PDB) [4]. Of its currently 50,000 structures, more than 40,000 have been determined using this method. Even though it is a very mature techology, certain ambiguities remain in the obtained structures. Particularly the resolution makes it difficult to detect hydrogen atoms [5], which must therefore be added in the following refinement of the data. We have modeled four degrees of freedom to predict positions of hydrogen atoms in protein structures:

### *Tautomeric states, especially in histidine*

Two different tautomeric states can be observed for histidine residues. Also, the hydrogen atom at carboxyl groups may change its binding partner.

### *Torsional angle changes*

In hydroxyl, thiol and amine groups, the orientation of the hydrogen atoms is not fixed. Due to the low energy barrier, they may rotate freely around the bond that connects the group to the rest of the molecule

*Protonation states *have been modeled for four functional groups: Groups that may carry a negative charge by losing one hydrogen include carboxyl and thiol residues, and groups that may carry a positive charge by taking an additional hydrogen atom charge are amine and imidazol groups.

### *Side-chain flips*

The identity of atoms in amide groups as well as in imidazole rings is hard to determine at common resolutions for protein crystal structures. Hence they may be rotated by 180° with respect to the PDB file's coordinates. In the following we will refer to the rotation of a functional group by by 180° as "side-chain flips".

As for the ligand, we only take into account changes of the torsional angle at hydroxyl, thiol and amine groups.

Several approaches addressing hydrogen placements in crystal structures have been developed. A thorough review can be obtained from Forrest and Honig [6]. Besides programs which calculate the position of hydrogen atoms by molecular dynamics minimization or place them solely by geometric criteria, the most frequently applied are WHAT IF [7], MolProbity [8], and the Hbuild [9] procedure implemented in the X-PLOR package. The programs differ mostly in their objective function and their optimization method. Hbuild uses the CHARMM force field to evaluate the quality of the formed hydrogen bonds, whereas WHAT IF features an empirical scoring function for hydrogen bonds. MolProbity on the other hand, uses "contact-dot" surfaces to model favorable interactions. The programs' optimization procedures can be grouped grossly in three categories. *Stochastic*, such as the simulated annealing used in WHAT IF, *greedy *as used in Hbuild and *exhaustive search*, as used in MolProbity.

Recently, two further methods have been published. The first is the Computational Titration algorithm [10] which uses a lightweight forcefield with the concept of "hydropathic interactions" as its objective function and an exhaustive enumeration for optimization. The second and most recent method is Protonate3D [11], which chooses the optimal states according to a chemical model derived from the MMFF94 [12] force field. It applies an exhaustive search on all combinations of admissible states of chemical groups and limits the search space by a prioritization of favorable states.

In this paper we describe Protoss, a new and fast method to calculate hydrogen atom positions based on optimal hydrogen bond networks. In contrast to the previously mentioned programs, our approach differs in two aspects: On the one hand, we ensure on the speed of the calculation by using an efficient dynamic programming approach with "memoization" [13], i.e. storing partial solutions and combining them to globally optimal solutions. On the other hand, we wanted to model the protein-ligand interface with an established method. Our objective function is based on the hydrogen bonding term of the Boehm scoring function [14] which has been designed to correctly reflect protein-ligand interactions and is used in the FlexX [15] molecular molecular docking program.

In the results section, a validation based on a dataset from Forrest and Honig is given. We demonstrate that we were able to reproduce the results with a quality that is comparable to that of the programs in a fraction of time, making the method suitable for high-throughput modeling applications.

## Results

### Algorithm

The algorithm starts by identifying hydrogen bond networks in the protein-ligand interface. In our context a hydrogen bond network is the maximal set of functional groups for which alternative modes exist and that are able to form hydrogen bonds among themselves. The networks are modeled as graphs: Every modeled degree of freedom is represented by a node, i.e. for each amino acid for which rules exist, included water molecules and all functional groups of the ligand that are treated. Edges stand for interactions between amino acids. Every node is assigned a set of admissible modes, which come from a set of pre-defined rules. The problem is now to find the modes in each network that yield the best hydrogen bond network with respect to our objective function. This can be done efficiently with a dynamic programming approach. The Protoss algorithm is split into two phases, initialization and optimization. The initialization is performed only once for a protein-ligand complex. The generated information can be used for alternative docking poses.

#### Initialization

The first step of the algorithm is to create modes for each residue for which rules exist (Table 1). Naturally, protonation states and side-chain flips are discrete. Rotational degrees of freedom as in hydroxyl or amino groups are approximated by using 12 discrete states with equally distributed torsion angles. For water, 60 orientations are generated, with its hydrogen atoms equally distributed in space. Water molecules have to be included explicitly by the user because of the problem of the distinction of bridging water molecules in the active site from solvent water which our method does not handle. In the ligand, only rotatable groups are considered, i.e. only hydroxyl, amine and thiol groups.

**Table 1:** Amino acids (and water) with their modeled degrees of freedom.

Amino Acid	Degrees of Freedom	#Modes
Asparagine	Flip state	2
Aspartic Acid	Protonation	3
Cysteine	Protonation, dihedral angle	13
Glutamine	Flip state	2
Glutamic Acid	Protonation	3
Histidine	Flip state, protonation	6
Lysine	Protonation, dihedral angle	18
Serine	Dihedral angle	12
Threonine	Dihedral angle	12
Tyrosine	Dihedral angle	12
Water	Both hydrogen atoms	60

**Figure 1:**
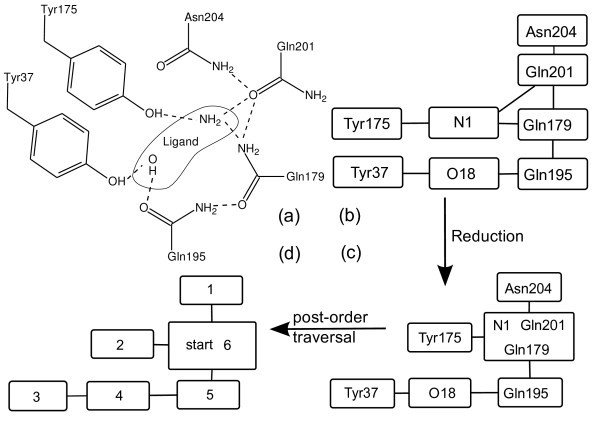
**A network from PDB structure **1x8x. This figure illustrates the algorithm that is used for optimization. The amine and the hydroxyl group of the ligand are included in the optimization. (a) A 2d-representation of the protein-ligand interface. (b) The graph representation of the hydrogen bond network. (c) Graph after reduction to a tree. The most central node is the root. (d) A depth-first traversal of the tree determines the order in which the nodes are evaluated. The numbers indicate the sequence of score calculation.

After all alternative modes have been generated, the nodes are connected such that the resulting graph represents the hydrogen bond networks in the protein's active site: Two nodes are connected with an edge, if any of their modes are able to form a hydrogen bond. A hydrogen bond network and the resulting graph for PDB structure 1x8x[16] is depicted in Figure 1.

Every mode is annotated with a base score which is its interaction score with the atoms that are not considered in the graph. Also, a small penalty of -0.1 is assigned to flipped modes and modes that represent uncommon protonation states, i.e. protonated carboxylates and unprotonated amines to ensure that the original version is retained if only minimally better interactions exist in an alternate mode (Table 2). After the graph has been built, it is divided into its connected components. Each component can be evaluated independently from the others. Components that consist only of a single node are trivial. Every mode is tested and the one resulting in the highest score with its environment is selected. For larger components, a more elaborate calculation has to be performed. Similar approaches have been implemented by Hooft et al. in the "cutting" procedure of WHAT IF [7] and by Canutescu et al. for the determination of chain rotamers in proteins [17].

**Table 2:** Overview of penalties assigned to modes.

Mode	penalty
Flipped group	0.1
Protonated carboxyl group	0.2
Positively charged histidine	0.1
Uncharged lysine	0.1

#### Optimization via dynamic programming

The first step of the optimization is to transform the graph into a tree. The most central node in the graph is chosen as the root of the tree (although theoretically any node could be chosen). Then, all biconnected components in the graph are replaced by single nodes. These new nodes inherit all edges of the nodes that it replaces and keeps references to all nodes that are part of the biconnected component.

The problem of finding the modes of amino acids that yield the best hydrogen bond network can be solved with a recursive procedure. An algorithm in pseudocode is given in Algorithm 1.

### Algorithm 1 – Dynamic programming procedure used in Protoss

The global array "sub" is used for memoization, the array "penalty" contains penalties for uncommon modes. "static_atoms" contains all atoms that have only one mode. Every mode has a unique identifier "id". The function score returns the interaction score of two modes. The attribute "toParent" of cycles denotes the node which was connected to the parent in the unreduced graph. The best modes are found via backtracking, which is not elucidated here for clarity reasons.

1: **function **CALC_SUBTREE(TreeNode N)

2:    **for **C **in **N.children **do **   ▻ Skipped if N is leaf

3:       CALC_SUBTREE(C)

4:    **if **N.isCycle **then**

5:       DECOMPOSE_AND_SOLVE_CYCLE(N.cycle)   ▻ Fills sub for N.cycle.toParent

6:    **else**

7:       **for **n **in **N.modes **do**

8:          sub [n.id] = SCORE(n, static_atoms) + penalty [n.id]

9:          **for **C **in **N.children **do **   ▻ Skipped if N is leaf

10:             best = -∞

11:             **if **C.isCycle **then **

12:                **for **c **in **C.cycle.toParent.modes **do **

13:                   best = max(best, SCORE(n, c) + sub [c.id])

14:          **else**

15:                **for **c **in **C.modes **do**

16:                   best = max(best, SCORE(n, c) + sub [c.id])

17:             sub [n.id] + = best

18: **function **FIND_BEST_SCORE(Tree T)

19:    CALC_SUBTREE(T.root)

20:    best = -∞

21:    **for **r **in **T.root.modes **do **

22:       best = max(best, sub [r.id])

23:    **return **best

The tree is traversed in a post-order fashion. For every tree node that is visited, the optimal solution of the subtree that it forms is computed and recorded. This is done by calculating two values for every mode: The first value is the interaction score of this mode with the atoms that have only a single mode, i.e. the atoms that are not changed throughout the procedure plus an optional penalty. The second value is summed over all children. For every child of the node, the maximum of the interaction scores with the current mode plus the maximum score of their corresponding subtrees is computed. The two values are added and recorded as the best achievable score for the current mode (lines 9 to 17). Because of the post-order traversal, the scores for the subtrees of the children have always been computed when the corresponding parent node is visited.

If more than one mode is found to yield an optimal score for one node, the mode that is equivalent to that in the PDB-file is chosen in case of protonation states, tautomers and side-chain flips. In nodes that represent rotational degrees of freedom, the median of all optimal modes that represent consecutive angles is chosen.

#### Decomposition of cyclic dependencies

A circular dependency does not allow for an application of the efficient dynamic programming procedure. Theoretically, all combinations of modes of its members would have to be evaluated. This quickly becomes infeasible because of the combinatorial explosion. Hooft et al. coped with this problem by using a simulated annealing procedure to find a good (but not necessarily the best) combination of modes.

Canutescu et al. limit the complexity of the problem with a branch and bound method. In Protoss we introduce a new concept to find the best scoring set of modes for the cyclic subgraphs.

We decompose cyclic dependencies by removing selected nodes from a compound until there are no cycles left in the graph. While all combinations of modes have to be tested for the removed nodes, the now acyclic part of the subgraph can be handled with the dynamic programming algorithm.

The cycle decomposition is conducted with a depth-first search. If a backedge is encountered during the search, the node of the cycle that has the fewest modes is removed and the search is restarted. This is repeated as long as the graph contains cycles. After all cycles have been decomposed, the dynamic programming algorithm is applied to the remaining non-cyclic parts for each combination of modes for the removed nodes.

### Testing

We tested the Protoss algorithm in two scenarios. First, we compared our predicted hydrogen positions with those in high-resolution protein structures which were able to determine hydrogen atom positions. Second, we conducted an analysis of so called NQ-flips in the Astex [18] dataset. NQ flips denote the wrong assignment of oxygen and nitrogen atoms in amides.

#### Hydrogen position prediction

In order to evaluate the quality of Protoss, we applied it to a test set published by Forrest and Honig [6] to assess the accuracy of programs which correct hydrogen atom positions in proteins. The test set consists of 34 hydrogen atoms from seven protein structures, which have either a resolution ≤ 0.9Å if they were obtained by X-ray crystallography or a resolution of ≤ 1.8Å if they were obtained by neutron diffraction. The hydrogen atoms have been selected automatically based on the program surfv [19], and are included, if their predicted solvent accessible surface is zero.

Furthermore, Forrest and Honig define a subset of eight hydrogen atoms for which they were able to confirm the positions by visual inspection of the electron density maps. They are all from a manually refined structure of the xylose isomerase (1muw; Fenn, Ringe, Petsko: unpublished work) [20]. In the following, we adopt the nomenclature used in the publication of the test set and call the complete set the "Buried" set and the subset the "Density" set.

**Figure 2:**
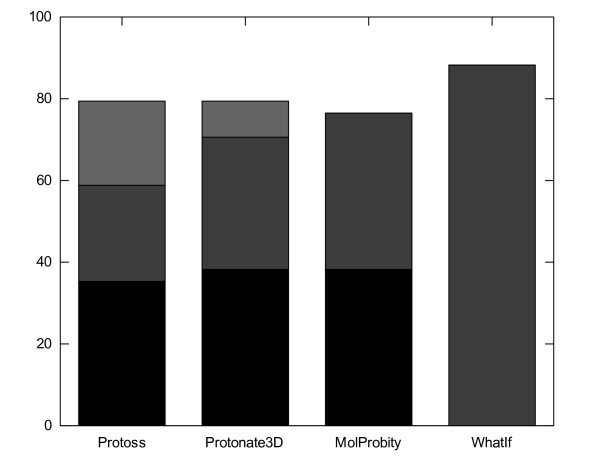
**Results for the Buried set in comparison**. The chart depicts the percentage of correctly placed hydrogen atoms. Colors: black < 0.2 Å, dark gray < 0.4 Å, light gray < 0.6 Å. The cutoff thresholds as well as the values for WhatIf and MolProbity were taken from Forrest [6]. Protoss as well as Protonate3D were run without the inclusion of water molecules.

We stripped all hydrogen atoms from the original PDB files with the program Reduce [8] to avoid a dependency on the original hydrogen positions, and let the FlexX standard routines add the missing hydrogen atoms. Then, we optimized the hydrogen bond networks with Protoss. Figure 2 shows that our method yields results that are comparable to that of the other programs. Out of the 34 hydrogen atoms in the Buried set, 10/20/27 are placed correctly at thresholds of 0.2/0.4/0.6 Å

**Figure 3:**
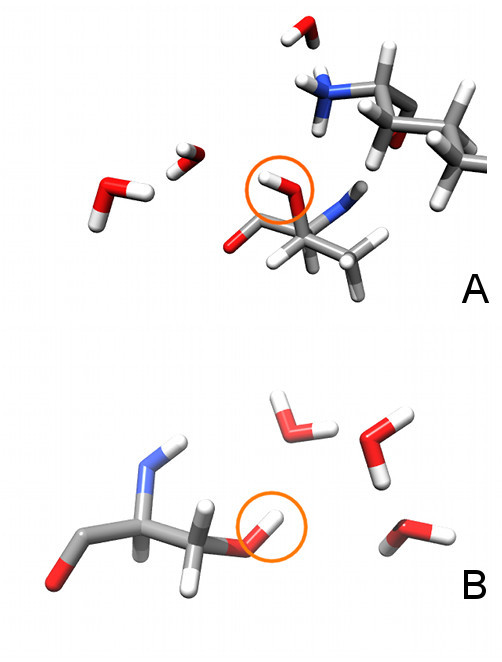
**Exposed residues in the Buried set**. Two examples of residues that are contained in the Buried set, but have contact to solvent molecules. A: Thr40 (Lys1 in top rightern corner), B: Ser91. Both examples are taken from PDB structure 1lzn[21].

**Figure 4:**
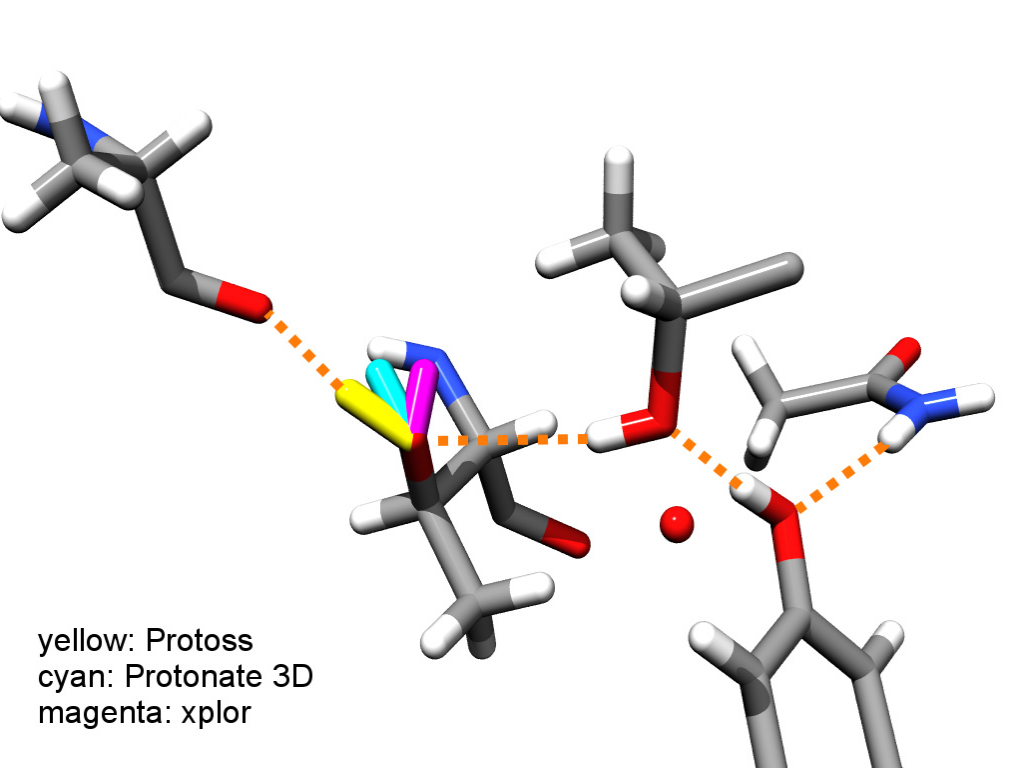
**Different predicted hydrogen atom positions**. Prediction of the hydrogen position of Thr118 by three Different programs in PDB structure 1muw. The structure is part of the Buried test set. Amino acids from left to right: Ala114, Thr118, Thr90, Thr133, Asn121. Protoss and Protonate3D orientate the hydrogen correctly, while X-PLOR is off by about an Angstrom.

In order to explain the inability to place all hydrogen atoms correctly, we conducted a visual check of both test sets. The check of the Buried set revealed that despite the selection procedure, four of the seven hydrogen atoms whose predicted position was off by more than 0.6 Å were exposed to the solvent, or belonged to hydrogen bond networks that extend to the solvent. Two examples where this is the case can be seen in Figure 3. In contrast to the core of the protein, exposed residues are much more mobile, and the prediction of one single "correct" hydrogen bonding pattern, may not be optimal. Instead describing different hydrogen bonding patterns that are equally valid, would be more appropriate. Apparently we have predicted one of the correct patterns, which happened to be different from that in the resolved structure. Another atom that was off is the hydroxyl hydrogen atom of Thr118 in 1muw. Protoss as well as Protonate3D position the hydrogen atom differently to that of the reference structure that was refined by X-PLOR (Figure 4). Visual inspection of the electron density maps and the protein structure showed that the positions of Protoss and Protonate3D are in better compliance with the electron density and better in terms of the hydrogen bond geometries. All 8 hydrogen atoms of the Density set lie within 0.4 Å of the reference atoms.

#### Amide flip prediction

Apart from the rotation of functional groups, Protoss also predicts so-called NQ-flips in protein structures as well as histidine flips. An NQ-flip denotes the wrong assignment of the oxygen and nitrogen atoms in the amide groups of asparagine (N) and glutamine (Q). Histidine residues may be flipped by 180° due to wrong assignments of carbon and nitrogen atoms in the imidazole ring. These flips are a frequently occurring ambiguity in PDB files, and result from the inability to distinguish certain heavy atoms in X-ray crystallography at common resolutions. A thorough statistical study on this matter by Weichenberger and Sippl [24] shows that about 21.0% of all amides in their test set would have more favorable interactions if they were flipped. Other figures lie in the same range: McDonald and Thornton(15%), Word (20.5%) [25] and Hooft (18%) [7].

We ran Protoss on the Astex dataset [18] which features a diverse set of protein structures with their associated ligands. Out of 4066 amino acids with amide groups in these proteins, 740 (18.2%) were predicted to be flipped, which is in the same range as the aforementioned analyses.

However, a following visual inspection revealed that the vast majority of the flipped groups lie on the surface of the proteins. Since amide groups are very polar, this is not very surprising. It does however limit the relevance of the amide flips to protein-ligand docking. Only 106 (2.6%) of the flips were found to be buried in the protein and less than 20 (< 0.5%) near an active site.

**Figure 5:**
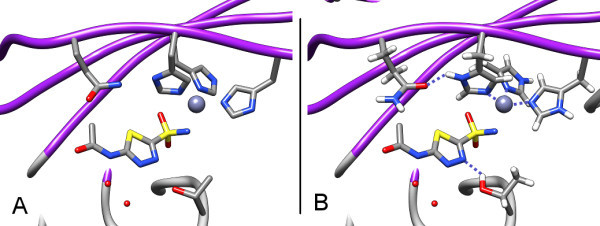
**An amide flip in an active site**. Amide flip prediction in the B-chain of PDB structure 1jd0[22]. In front is the ligand, from left to right: Gln92, His94, His96, His119, on the bottom is Ser200. A: Structure as in the PDB. B: Automatically corrected structure. The histidines are correctly protonated to interact with the metal ion, and the amide group in Gln92 is flipped to allow for a hydrogen bond with the N_*δ *_of the leftmost histidine. Non-polar hydrogen atoms were added by standard FlexX routines.

We selected three successful examples of the the automated flip detection in Protoss, where the flips occur close to the active site. Protein structure 1jd0 contains a flipped glutamine residue. As can be seen in Figure 5, Gln92 is rotated by 180 degrees to allow for an interaction with His94, which again coordinates a metal ion.

**Figure 6:**
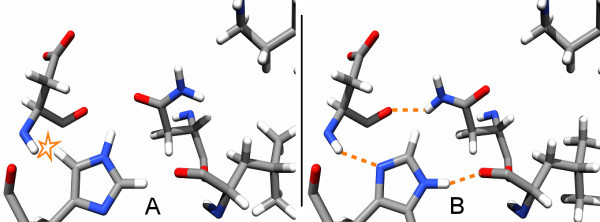
**Histidine flip prediction in an active site**. Histidine flip prediction in structure 1ywr[23]. From left to right: Asp150, His148, Asn155, Leu167. A: Structure as in the PDB. B: Automatically corrected structure. In the top rightern corner is a part of the cocrystallized ligand. The original structure results in one atom clash for His148 and several unsatisfied hydrogen bonding groups. Protoss rotates the histidine to allow for two hydrogen bonds with the backbone atoms of Asp150 and Leu167. Also, Asn155 is flipped to allow for a favorable hydrogen bond with Asp150.

For protein structure 1ywr two flips are predicted. His148 is rotated by 180° to allow for two new hydrogen bonds and to avoid a clash with Asp150. Also, Asn155 is flipped to allow for a new hydrogen bond with the backbone of Asp150 and to move the two negatively charged oxygen atoms away from each other (Figure 6).

Another flipped side chain occurs in the active site of 1ig3. The PDB conformation in Asn239 would result in atom clashes and a non-optimal hydrogen bond network. If it is flipped, it could interact with Ser236 which in turn interacts directly with the ligand. This is especially important since flipping Asn239 and allowing it to take part in the hydrogen bond network is needed to correctly assess the interaction with the ligand.

### Time usage

After the protein-ligand complex is read in and prepared by the FlexX library routines, and the interaction surfaces are assigned, two phases can be distinguished. First, the alternative modes are generated and the hydrogen bond networks are identified. Second, the optimization procedure to find the best hydrogen positions is conducted.

**Figure 7:**
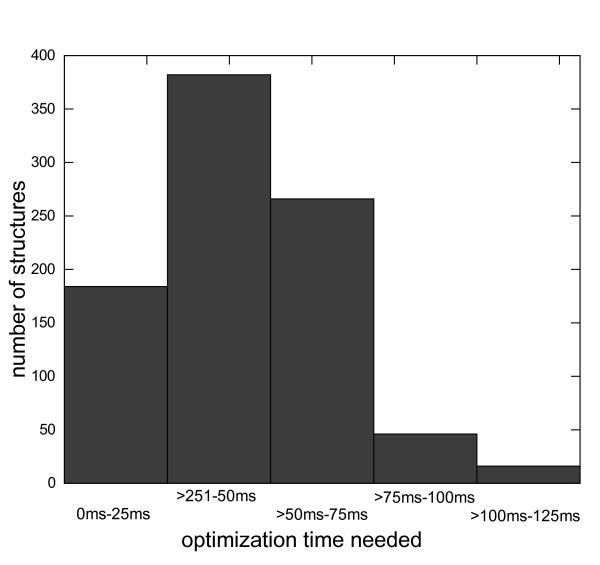
**Time consumed by optimization of a complex**. 900 active sites from the PDBbind dataset were analyzed. 6 took longer than 0.125 seconds. Only the time for the optimization phase is considered. The complexes are binned with respect to the time that was needed to optimize them.

To time the program, 900 active sites and their corresponding ligands of the PDBbind [26] dataset in the version of 2004 were extracted (all amino acids within 6.5 Å of any ligand atom) and optimized. The overall time needed to process all complexes was 9 minutes and 44 seconds (0.6s per active site) on one 2.66 Intel Xeon CPU with SUSE Linux 10.2. For the second phase, i.e. the optimization alone, only 38 seconds (0.04s per active site) were needed. A more detailed analysis, as depicted in the histogram in Figure 7, reveals that most active sites can be corrected in less than 0.125 seconds. Only 6 active sites needed more time than that, apparently because they have large circular dependencies, which could not be decomposed well.

In a scenario, where repeated optimizations of the hydrogen bond network are conducted, the relatively slow first phase has to be carried out only once. The speed of the second phase however would allow for a fast optimization of the hydrogen bond network in consecutive calls. This scenario might be an adjustment of hydrogen atoms prior to a detailed scoring function evaluation of a protein-ligand complex. This is frequently encountered in docking calculations, where this step has to be done multiple times to evaluate predicted poses.

### Limitations

#### Strongly interconnected compounds

**Figure 8:**
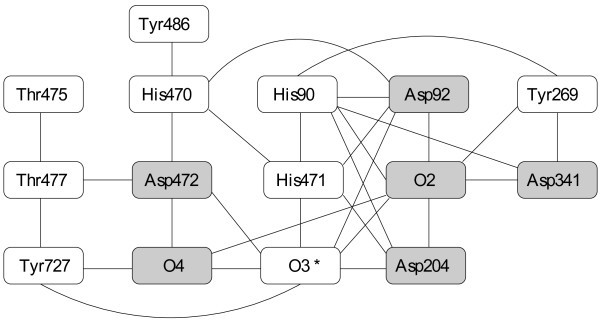
**Example of a hydrogen bond network**. Hydrogen bond network in the active site of PDB structure 1ps3 as perceived by Protoss. O2, O3 and O4 are hydroxyl atoms in the ligand. The gray nodes are the nodes that are removed by the cycle decomposition algorithm. The node with the star is the root of the tree traversal.

The dynamic programming approach of Protoss does not work well on strongly interconnected graphs such as in protein structure 1ps3[27] which is contained in the PDBBind dataset. The total time needed to optimize the complete protein is 9.5s, of which the optimization procedure alone takes 2.22s. This is still fast compared to other methods such as Protonate3D, which takes approximately 220s for the complete process and 83s for the optimization, but takes much longer than most other proteins. The optimization time is dominated by an extensive hydrogen bond network that spans over 15 residues and needs 0.75s to be solved. A sketch of the hydrogen bond network is provided in Figure 8. Protoss has to test 1.1·10^6 ^combinations of modes for the removed nodes. Despite being relatively slow, it is a huge reduction since the search space for all combinations of modes is in the order of 7.5·10^12^.

The time could be reduced by ignoring side chain flips and applying constraints on when an edge is inserted into the graph, i.e. apply a minimum quality threshold on the considered hydrogen bonds.

#### Further degrees of freedom

In principle, further degrees of freedom influence the hydrogen bond networks. In ligands, tautomeric and/or protonation states may change in response to the protein. Also, interfacial water molecules can be part of the networks. In its current form, Protoss does not consider different protonation states of the ligand or tautomeric forms in the ligand. The method is however capable of including these additinal degrees of freedom. And although Protoss can orient selected water molecules, it can not yet predict the presence of water molecules in the protein-ligand interface. Protonation, tautomers as well as interfacial water molecules are of importance for the prediction of protein-ligand complexes and are therefore an interesting direction for further research.

#### Predicting stability and alternative hydrogen bonding networks

Our method gains its speed from considering only the best scoring solutions. However, quite different solutions with a similar score might exist. This would for example be the case in active sites of enzymes which feature a catalytic triade where a proton is transferred onto a substrate. Both states, the one before and the one after the transfer of the proton, have valid networks that can both be encountered. Instead of obtaining only the best scoring solution, it would be desirable to obtain a set of stable conformations that are valid for the active site. Since Protoss is intended to be applied on different ligands and and poses individually upon molecular docking and scoring, alternative hydrogen bond networks are of less importance. Nevertheless, an extension in this direciton is methodologically feasible.

### Implementation

The method has been implemented in ISO-C using parts of the FlexX [15] source code. It is not available as a stand-alone version but will be used as a basis for protein preparation in further projects such as in two-dimensional protein-ligand depiction (Poseview [28]), re-scoring (HYDE [29]) and in the preparation of active sites for protein-ligand docking with FlexX.

## Conclusion

We have implemented a program that automatically places hydrogen atoms in protein structures with particular focus on protein-ligand interfaces. Having that information is important for any subsequent calculations, particularly in structure-based design approaches to finding new ligands.

The prediction of the positions of hydrogen atoms is consistent with those in the test sets of Forrest and Honig, and the reported rate for amide chain flips is in unison with the rates reported in the literature. In the Density set, all hydrogen atoms are placed within 0.4 Å. For a lower tolerance, i.e. 0.2 Å, this quota is slightly worse. A placement within 0.4 Å however suffices for the FlexX scoring function, as it is robust enough to compensate for inaccuracies of this order. In fact, the inaccuracies can be traced back to this robustness. The important fact is that the hydrogen atoms are placed facing into the right direction, thus making it possible to correctly identify any interactions that they are involved in.

The novelty in this method is that it always finds a maximum score solution with respect to our objective function for all hydrogen positions in a hydrogen bond network, whereas previous methods usually tackled the large search space by resorting to heuristic strategies such as greedy algorithms or stochastic search methods. Our method typically takes less than a tenth second to optimize a hydrogen bond network in an active site, even if it contains cyclic dependencies. However, if cyclic dependencies exist that cannot be decomposed well, this time might be exceeded.

One open question is how to model hydrogen bonds that are formed with bulk solvent molecules. Since water molecules may act both as an acceptor and as a donor and are constantly moving, it is a difficult task to correctly identify and assess hydrogen bonds that may be formed. In summary, we believe that the Protoss method is a useful component for all software tools that model protein-ligand complexes.

## Experimental

### Scoring function

Protoss needs to evaluate the quality of the formed hydrogen bond networks. For this task it uses the interaction model that has been incorporated in the docking program FlexX [15] and which is based on an empirical scoring function genuinely developed by Boehm [14].

**Figure 9:**
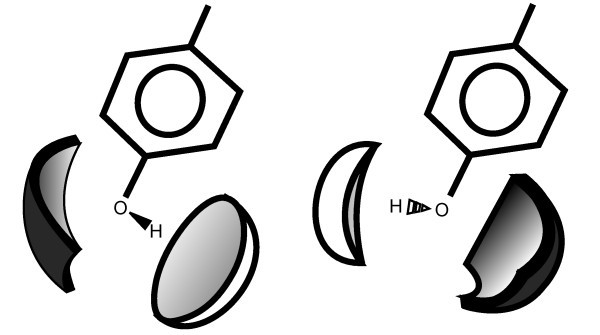
**Interaction surfaces**. Interaction surfaces of two rotamers of tyrosine. The dark interaction surface encodes the hydrogen bond acceptor capability, the light surface encodes the hydrogen bond donor capability. Depending on the position of the hydrogen atom, the spatio-chemical properties of the group change.

Interactions are modeled by interaction surfaces. These surfaces are assigned to a molecule based on its functional groups and geometric properties. For example, every hydroxyl group has two interaction surfaces which represent the ability to form hydrogen bonds: One for the ability to act as a hydrogen bond donor in the direction of the hydrogen atom, and another for the ability to act as a hydrogen bond acceptor in the direction of the free electron pairs (see Figure 9 for an example). A pair of compatible functional groups contributes to the calculated free energy of the protein-ligand complex, if the geometric properties encoded in the partial spherical interaction surfaces obey the constraints of the scoring function. Ideal geometries for interacting groups are described using an optimal distance and a bond angle for both interaction partners. This term results in a score between 0.0 and 1.0 for each interaction, where 0.0 corresponds to a non-existing interaction and 1.0 to an ideal geometry.

The score for an interaction is calculated by evaluating the following equation for three descriptors: the deviation Δ from the ideal distance and the deviation from the ideal bond angles for each of the interaction partners.

Here, Δ is the deviation from the ideal value, *d*_1 _and *d*_2 _describe the tolerance towards deviation from the ideal geometry and depend on the functional groups that interact. The three values are multiplied and taken as a measure for the quality of the formed interaction.

In Protoss we restrict the chemical model to the geometric properties of hydrogen bonds and metal interactions. The optimization finds the set of modes that has a maximal number of interactions considering possible penalties. The score for one set of modes is calculated by:

such that interactions(*m*) is the sum of hydrogen bond scores as described above and penalty(*m*) the penalty for flipping groups or choosing uncommon protonation states as listed in Table 2.

### Decomposition of cyclic dependencies

When a cycle is encountered in the dependency graph, nodes are removed until the graph becomes acyclic. Determining if a subset *V' *⊆ *V *exists with |*V'*| ≤ *k *for a positive integer *k *is known as the Vertex Feedback Set problem, which is NP-hard [30]. In our context the graphs are relatively small. Therefore we can rely on a greedy heuristic to minimize the number of times the remaining graph has to be traversed. This is important since the traversal has to be conducted for each combination of modes in *V'*. The algorithm applied here iteratively searches for cycles. Once a cycle is found, the node with the minimal number of modes becomes part of the subset.

### Sampling of water orientations

In order to model the many possibilities that exist for the orientation of water molecules, we sample 60 orientations. We use an icosahedron that is centered at the position of the oxygen atom to ensure an equal distribution of the individual samples. An initial placement is created by orientating the water molecule such that the angle bisector of its opening angle points to one of the corners of the icosahedron. Then an orientation is created for each of the five corners that lie opposed to the first corner: The coordinates of the water's hydrogen atoms are chosen such that they lie in a plane with each of the second corners. This results in five orientation for each of the twelve corners, totaling up to 60 orientations for a water molecule.

## Competing interests

BioSolveIT GmbH, which has been co-founded by MR, has financially supported TL for the work on this project.

## Authors' contributions

TL developed, implemented and tested the presented method. TL prepared the manuscript for this publication. MR supervised and coordinated the project. Both authors have read and approved of the final manuscript.
